# Subcutaneous metastasis of rectal adenocarcinoma: an unusual and fatal metastatic localization

**DOI:** 10.11604/pamj.2022.42.211.35282

**Published:** 2022-07-18

**Authors:** Tarik Souiki, Khalid Mazaz

**Affiliations:** 1 Faculty of Medicine and Pharmacy, Sidi Mohammed Ben Abdellah University, Fez, Morocco,; 2 Department of Visceral Surgery E3, University Hospital Hassan II, Fez, Morocco

**Keywords:** Rectal adenocarcinoma, subcutaneous metastasis, resection

## Image in medicine

Subcutaneous metastasis of colorectal cancer is extremely rare. It is typically associated with a poor prognosis. Herein, we present a case of a 38-year-old young woman, diagnosed with stage III of rectal adenocarcinoma. She received neoadjuvant chemoradiation and was scheduled for total mesorectal excision after eight weeks of delay. During the week preceding surgery, she complained of the occurrence of two subcutaneous nodules, the first one in the abdominal wall, at the left hypochondrium, and the second in the low part of the chest wall. The physical examination showed well-defined, mobile, tender subcutaneous nodules without epidermal changes. The thoracoabdominal-pelvic computed tomography (CT) scan showed two enhanced nodules localized in the abdomen (A) and in the chest walls (B), measuring respectively 10 mm and 8 mm. Given the young age of the patient, it has been decided to perform mesorectal total excision associated with biopsy excision of subcutaneous nodules (C). The histological study of the rectal specimen showed a complete pathological response and safe margins (PT0N0). However, results in nodule specimens revealed poorly differentiated adenocarcinoma that was cytokeratin CK-20 and CDX2 positive (D). These results suggested a metastasis from the lower gastrointestinal tract. Unfortunately, the patient's general condition deteriorated rapidly in the first postoperative month. New subcutaneous nodules appear in the abdominal wall, the chin, and the cuisse. Besides, body CT and 18-Fluorodeoxyglucose positron emission tomography scans revealed disseminated disease including pancreas, adrenal, and peritoneum. The patient started chemotherapy but died after two cycles from a widely disseminated disease.

**Figure 1:**
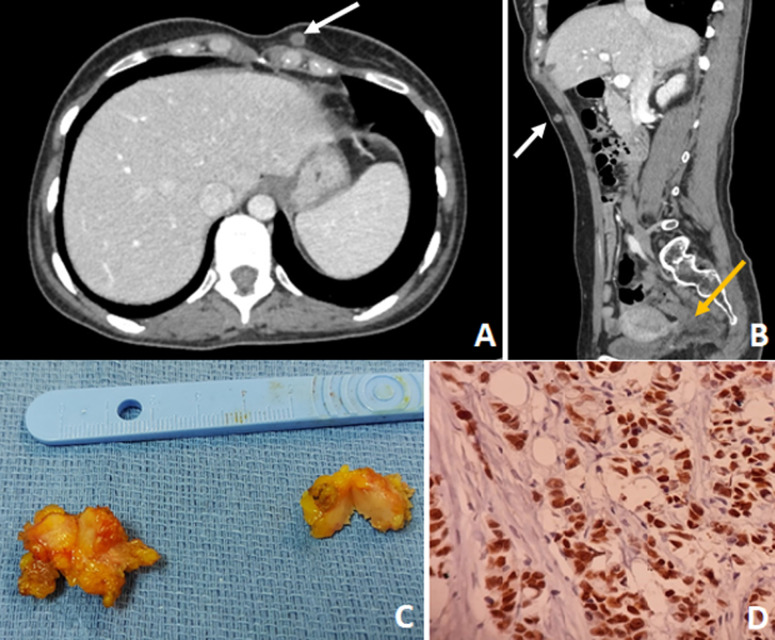
A) CT scan (axial view) showing a subcutaneous metastatic nodule of the chest wall (arrow); B) abdominal CT scan (sagittal view) showing the subcutaneous metastatic nodule of the abdominal wall (arrow) and tumoral rectal thickening (yellow arrow); C) resected specimen nodules; D) nuclear immunostaining for CDX2 (original magnificationx200)

